# Modulation of ovine SBD-1 expression by *Saccharomyces cerevisiae* in ovine ruminal epithelial cells

**DOI:** 10.1186/s12917-018-1445-9

**Published:** 2018-04-19

**Authors:** Xin Jin, Man Zhang, Xue-min Zhu, Yan-ru Fan, Chen-guang Du, Hua-er Bao, Siri-guleng Xu, Qiao-zhen Tian, Yun-he Wang, Yin-feng Yang

**Affiliations:** 1 Veterinary Medicine College of Inner Mongolia Agricultural University, Hohhot, 010018 People’s Republic of China; 2 Key Laboratory of Clinical Diagnosis and Treatment Technology in Animal Disease, Ministry of Agriculture, Hohhot, 010018 People’s Republic of China; 3 College of Animal Science and Technology, Henan University of Science and Technology, Luoyang, 471000 People’s Republic of China; 4 Vocational and Technical College of Inner Mongolia Agricultural University, Baotou, 014109 People’s Republic of China

**Keywords:** Sheep, Ruminal epithelium, *Saccharomyces cerevisiae*, SBD-1, Modulation, Signalling pathway

## Abstract

**Background:**

The ovine rumen is involved in host defense responses and acts as the immune interface with the environment. The ruminal mucosal epithelium plays an important role in innate immunity and secretes antimicrobial innate immune molecules that have bactericidal activity against a variety of pathogens. Defensins are cationic peptides that are produced by the mucosal epithelia and have broad-spectrum antimicrobial activity. Sheep β-defensin-1 (SBD-1) is one of the most important antibacterial peptides in the rumen. The expression of SBD-1 is regulated by the probiotic, *Saccharomyces cerevisiae (S.c)*; however, the regulatory mechanism has not yet been elucidated. In the current study, the effects of *S.c* on the expression and secretion of SBD-1 in ovine ruminal epithelial cells were investigated using quantitative real-time PCR (qPCR) and enzyme-linked immunosorbent assay (ELISA). In addition, specific inhibitors were used to block the nuclear factor kappa-light-chain enhancer of activated B cells (NF-κB), p38, JNK, and ERK1/2 signalling pathways separately or simultaneously, to determine the regulatory mechanism(s) governing *S.c*-induced SBD-1 upregulation.

**Results:**

Incubation with *S.c* induced release of SBD-1 by ovine ruminal epithelial cells, with SBD-1 expression peaking after 12 h of incubation. The highest SBD-1 expression levels were achieved after treatment with 5.2 × 10^7^ CFU∙mL^− 1^ *S.c.* Treatment with *S.c* resulted in significantly increased NF-κB, p38, JNK, ERK1/2, TLR2, and MyD88 mRNA expression. Whereas inhibition of mitogen-activated protein kinases (MAPKs) and NF-κB gene expression led to a decrease in SBD-1 expression.

**Conclusions:**

*S.c* was induced SBD-1 expression and the *S.c*-induced up-regulation of SBD-1 expression may be related to TLR2 and MyD88 in ovine ruminal epithelial cells. This is likely simultaneously regulated by the MAPKs and NF-κB pathways with the p38 axis of the MAPKs pathway acting as the primary regulator. Thus, the pathways regulating *S.c*-induced SBD-1 expression may be related to TLR2-MyD88-NF-κB/MAPKs, with the TLR2-MyD88-p38 component of the TLR2-MyD88-MAPKs signalling acting as the main pathway.

## Background

Defensins, a group of broad-spectrum antimicrobial agents secreted by epithelial cells in response to microbial infection and stimulation from the main components of microbial pathogens, have recently garnered a lot of attention. Defensins are small-molecular-weight peptides, generally consisting of 29–42 amino acids, which play an important role in both the innate and adaptive immune systems of vertebrates [1–3]. In contrast, pathogen defense in invertebrates and plants occurs exclusively via mechanisms involved in innate immunity [4, 5].

Defensins have a lethal effect on both gram-negative and gram-positive bacteria, as well as on viruses and fungi [6–8]. Defensins are divided into 3 main classes, based on different structural characteristics: α-, β-, and θ-defensins. To date, α- and θ-defensins have only been identified in mammals [6, 9]. The expression of α-defensin genes has been observed in humans [10], mice [11], rhesus macaques [12], rats [13], rabbits [14], guinea pigs [15], hamsters [16], and horses [17]. The α-defensins have also been identified in opossum [18], elephant, and hedgehog tenrec genomes in silico [19], but are absent from cattle [20] and dog genomes [21]. The θ-defensins have only been identified in the rhesus macaque (*Macaca mulatta*) and olive baboon (*Papio anubis*) [22]. The β-Defensins have been found in various vertebrates, including cows [23], humans [24], mice [25], birds [26], reptiles [27], and fish [28]. Thus far, only beta defensin-1 (BD-1) and beta defensin-2 (BD-2) have been identified in sheep [29]. In adult sheep, SBD-2 expression was found to be confined to the tongue, ileum, and colon, while SBD-1 expression was identified in the entire digestive tract (from the tongue to the colon), with highest expression located in the rumen [30, 31]. This suggests that β-defensins constitute an important component of ovine rumen innate immunity.

Animal feed producers have used specific strains of *S.c* as feed supplements, based on claims that these products can improve feed intake [32, 33], weight gain [34], fiber digestion [35, 36], and reduce the need for antibiotic use. It has also been reported that live yeast can stabilize the rumen pH [37, 38]. In addition to the nutritional value of yeast, there is evidence that yeast probiotics and components, such as zymosan, can increase the production of the host defense peptide, cathelicidin, and the cytokine, IL-1β, in the intestinal epithelial cell line RTgutGC, at the mRNA and protein levels [39]. In addition, whole yeast (*S.c*), β-glucan and laminaran have been used as immunostimulants in farmed Nile tilapia. The β-glucans have been used in farmed Nile tilapia under stressful, immune-depressive conditions, in order to increase resistance to disease [40]. Therefore, it is possible that dietary supplementation with yeast probiotics and/or their components may improve ovine innate immunity in animals that are vulnerable to disease.

Nonetheless, there is a paucity of information concerning the role and regulation of SBD-1 gene expression in the sheep rumen; particularly whether *S.c* can modulate SBD-1 expression. In this study, the expression of SBD-1 was investigated in ovine ruminal epithelial cells treated with *S.c* using qPCR and ELISA assays. In addition, the effect of the p38, ERK1/2, JNK, and NF-κB– pathways on SBD-1 expression in ruminal epithelial cell culture was examined. The results indicated that *S.c* provides a stimulus that may regulate defensins by MAPKs and NF-κB pathways.

## Methods

### Reagents

The NF-κB inhibitor, PDTC, the ERK1/2 inhibitor, PD98059, the p38 inhibitor, SB202190, and the JNK inhibitor, SP600125, were purchased from Sigma Chem. Co. (Munich, Germany). All other chemicals used were of analytical grade and obtained from commercial sources.

### Fungal strains and culture conditions

The *S.c* used in this study was purchased from the Chinese microbial strain network (code: CGMCC 2.161). The yeast strains were inoculated in 100 mL malt extract medium and incubated for 48 h at 28 °C in an orbital shaker (180 rpm).

### Ovine ruminal epithelial cells and culture conditions

Ten adult Mongolian sheep (5 ewes and 5 rams, aged 10–15 months) were obtained from Inner Mongolia Agricultural University (IMAU) Experimental Animal Center. None of the sheep had clinical signs of parasitic or infectious disease. The sheep were euthanized with an overdose of the proprietary euthanasia solution Euthasol (pentobarbital sodium 100 mg/kg and phenytoin sodium 10 mg/kg) and the rumens were harvested. Rumen epithelial cells were obtained from each of the 10 sheep and were tested separately. This study was approved by the Institutional Animal Care and Use Committee of the IMAU (License No. SYXK, Inner Mongolia, 2014–0008) with adherence to IMAU guidelines.

After euthanasia, the rumen tissue (25 cm^2^) was immediately extracted, flushed with physiological saline, and placed in ice-cold phosphate buffered saline (PBS; Sigma-Aldrich) supplemented with 5% penicillin/streptomycin (Sigma). The ruminal epithelial cells were cultured as previously described [41, 42]. All procedures were performed under sterile conditions. The tissue was washed several times with PBS and the mucosa was removed from the underlying epithelium and washed 3 times in PBS supplemented with 1 mg/mL penicillin, 500 μg/mL streptomycin, 100 μg/mL gentamicin, and 50 μg/mL amphotericin. Seven digestions of the ruminal mucosal tissue were performed with 0.25% pancreatin (Sigma) incubated at 37 °C for 45, 40, 30, 20, 15, 8, and 3 min; the digestion products were then observed under a microscope. A large number of small cells were observed after the third digestion that were predominantly oval or round in shape, had smooth edges, and high refractive indexes. Ruminal tissue was removed and the cell pellet was resuspended in DMEM containing 20% fetal bovine serum (FBS, approximately 20 mL) to stop enzymatic digestion and then concentrated by centrifugation at 1000 rpm for 6 min. The cell pellet was again resuspended in DMEM, agitated by pipetting up and down using a movette pipette 3–5 times, and cultured at 37 °C and 5% CO_2_ in DMEM/F12 supplemented with 20% heat-inactivated FBS, 200 μg/mL penicillin, 100 μg/mL streptomycin, 50 μg/mL gentamicin, 5 μg/mL amphotericin, 2 μg/mL insulin-protein- selenium additive, and 2 μg/mL β-mercaptoethanol. The cells remained attached to the cell culture plate for more than 96 h and the medium was replaced every 2 d. Once the number of primary cells grew to more than 85% confluence, the cells were passaged into 12-well flat-bottom culture plates.

### Induction tests

After reaching 80–90% confluence, the ovine ruminal epithelial cells were washed 3 times with PBS containing 1% penicillin/streptomycin and then passaged into 12-well flat-bottom culture plates containing 1 mL DMEM/F12 medium, without FBS or antibiotics, and were incubated at 37 °C and 5% CO_2_ for a 24 h starvation treatment. Following the starvation treatment, the cells were washed 3 times with PBS and placed into 900 μL DMEM/F12 without FBS or antibiotics. The cells were then randomly divided into 6 groups: 5 *S.c*-treated groups and 1 control group. The treatment groups were exposed to a range of concentrations (5.2 × 10^8^, 5.2 × 10^7^, 5.2 × 10^6^, 5.2 × 10^5^, 5.2 × 10^4^ CFU∙mL^− 1^) of *S.c* (100 μL) at 37 °C and 5% CO_2_ for 2 h; control group cells were cultured in DMEM/F12 medium without *S.c*. After 2 h of stimulation, the medium was discarded and the cells were washed 3 times with PBS containing 5% amphotericin and incubated for 2, 4, 8, 12, or 24 h in DMEM/F12 with antibiotics. Finally, total RNA was extracted from the cells following induction for 2, 4, 8, 12, or 24 h.

### Inhibition tests

The NF-κB, p38, JNK, and ERK1/2 pathways were blocked using specific inhibitors: PDTC (50 μM) for NF-κB, SB202190 (20 μM) for p38, SP600125 (20 μM) for JNK, and PD98059 (20 μM) for ERK1/2. The cells were treated with the inhibitor for 1 h and the 4 different treatment groups were then stimulated with *S.c* (*S.c* + PDTC, *S.c* + PD98059, *S.c* + SB202190, or *S.c* + SP600125). Cells treated with inhibitors (PDTC, PD98059, SB202190, SP600125), but not *S.c*, were the negative controls. Cells treated with *S.c*, but not inhibitors were the positive controls and untreated cells were the blank controls.

### Primers

Primers used to detect the expression of target genes (SBD-1, TLR2, MyD88, NF-κB, p38, JNK, ERK1/2) in ovine ruminal epithelial cells treated with *S.c* are shown in Table 1. The reference gene (β-actin) in the current study was used as a reference gene in a similar previous study [43, 44]. Stable expression of this gene was validated using PCR and Western blot. All primers (Table 1) were designed and synthesized by Sangon Biotech (Shanghai, China).

**Table 1:** Primer sequences for qPCR

Gene names	GenBank accession	Fragment size (bp)	Primer pair sequences (5`-3`)
SBD-1	U75250	206	F: GGCTCCATCACCTGCTCCTC
			R: CGTCTTCGCCTTCTGTTACTTCTT
β-actin	U39357	208	F: GTCACCAACTGGGACGACA
			R: AGGCGTACAGGGACAGCA
TLR2	DQ890157.1	190	F: GTGTCCGCCGTGTGCTGTGC
			R: AGTAGGAATCCCGCTCGCTGTAGG
MyD88	GQ221044.1	203	F: AGGTGCCGTCGGATGGTGGTGGTT
			R: TGGTGGCAGGGGTTAGTGTAGTCA
NF-κB	XM_012119628.1	95	F: CACCTTCTCCCAGCCCTTTG
			R: TGCCACCTCCTCCTCCAG
p38	NM_001142894.1	74	F: CGTTCAGTTCCTTATCTACCAG
			R: GCTCACAGTCTTCATTCACAG
JNK	XM_004002020.3	113	F: ATGACTGCAAAGATGGAAACGA
			R: ATGCTCTGCTTCAGAATCTTGG
ERK1/2	XM_012157699.1	90	F: GCGCTACACCAATCTCTCGT
			R: ATGGCGACTCGGACTTTGTT

### RNA isolation

After reaching 85–95% confluence in 12-well flat-bottom plates (~ 450,000 cells per well), the epithelial cells were washed with PBS. Total cellular RNA was extracted using RNA Fast2000 (Fastgene, China), in accordance with the manufacturer’s instructions. The total RNA concentration and purity were analysed by measuring the absorbance at 260 and 280 nm using a Synergy H4 Hybrid microplate reader (BioTek Inc., Winooski, VT, USA). Only samples with an OD 260/280 ratio between 1.8 and 2.0 were included in subsequent studies.

### cDNA synthesis

RNA was reverse transcribed into cDNA using the PrimerScript RT reagent Kit with gDNA Eraser (TaKaRa, Japan) via a thermal cycler (GeneAmp PCR System 9700, Thermo Fisher Scientific Inc., Massachusetts, USA). To remove genomic DNA, a total of 500 ng of RNA from each sample was first incubated at 42 °C for 2 min in the presence of 0.5 μL of RNase-Free dH_2_O, 1.0 μL of gDNA Eraser, and 2.0 μL of 5× gDNA Eraser Buffer. Next, reverse- transcription (RT) was conducted in a total reaction volume of 20 μL, containing 10 μL of extracted DNA template, 4.0 μL of RNase-Free dH_2_O, 4.0 μL of 5× Prime Script Buffer 2 (for qPCR), and 1.0 μL of Prime Script RT Enzyme Mix I. The thermal profile consisted of 37 °C for 15 min, 85 °C for 5 s, and the synthesized cDNA stored at − 20 °C. The absence of contaminating gDNA was confirmed by the absence of product in control tubes (no RT), which included all components of the cDNA synthesis reaction, except Prime Script RT Enzyme Mix I.

### qPCR

**Fig. 1:**
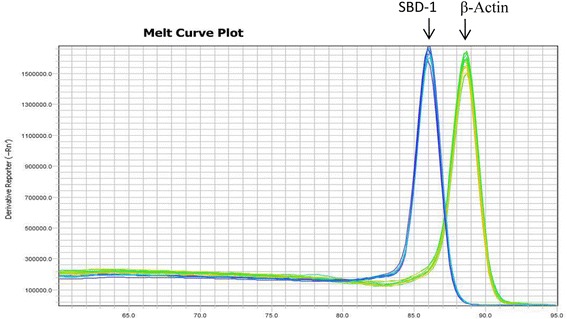
qPCR melt peak curve for SBD-1 and β-Actin genes. Primers are valid for qPCR as demonstrated by a single peak observed in each dissociation curve using SYBR Green II

Following reverse transcription, the cDNA samples were analysed by qPCR using the VIIATM7 Real-Time PCR System. Each 20 μL reaction was used for qPCR analysis in PCR strip tubes (PCR-0208-C, Axygen) and consisted of 10 μL of SYBR Premix Ex Taq (2×, Perfect Real Time, TakaRa), 0.8 μL of each gene-specific primer (10 μM), 2.0 μL of the cDNA template, and 6.4 μL of ddH_2_O, which was used to normalize the fluorescent reporter signal between the reactions. The reaction conditions were as follows: 95 °C (30 s); 45 cycles of 95 °C (5 s); 60 °C (34 s). Each condition was followed by a 46-step melt-curve analysis (95 °C for 5 s, 60 °C for 30 s, and 95 °C for 15 s). All PCR reactions were performed in triplicate and primer specificity was confirmed by melting (dissociation) curve analysis (Fig. 1). Gene-specific amplification efficiencies for primer were also measured. Quantification of relative expression of the target gene was performed using the 2^−ΔΔCt^ method and QuantStudio™ Real-Time PCR software.

### SBD-1 ELISA

Cell culture supernatants were collected to determine the secretion of SBD-1 by ovine ruminal epithelial cells upon induction with different concentrations of *S.c* (5.2 × 10^8^, 5.2 × 10^7^, 5.2 × 10^6^, 5.2 × 10^5^, 5.2 × 10^4^ CFU∙mL^− 1^) for 2, 4, 8, 12, or 24 h. The SBD-1 protein was quantified in the cell culture supernatants from ovine ruminal epithelial cells using the Ovine defensin β1 (DEFβ1) ELISA kit (Wuhan Xinqidi Biological Technology, China) according to the supplier’s protocol.

### Statistical analysis

All experiments were performed in triplicate and were repeated at least 3 times. The data are presented as the mean ± SD of 3 independent experiments. A 1-way analysis of variance (ANOVA) was used to compare the differences between the control and treatment groups using IBM SPSS Statistics 20.0 (SPSS Institute Inc.). The Duncan’s multiple range test was used to assess the differences after verifying normality and homogeneity of variance. *P* values of less than 0.05 were considered to be statistically significant.

## Results

### *S.c* induced SBD-1 mRNA expression in ovine ruminal epithelial cells

**Fig. 2:**
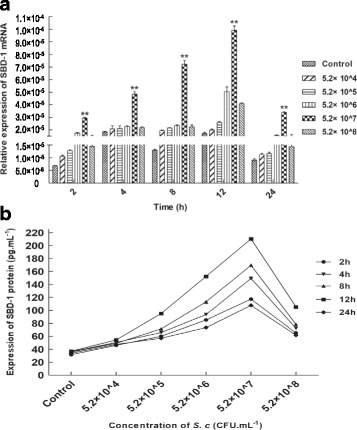
*S.c* induces SBD-1 mRNA and protein expression. **a** Ovine ruminal epithelial cells treated with *S.c* (5.2 × 10^8^, 5.2 × 10^7^, 5.2 × 10^6^, 5.2 × 10^5^, 5.2 × 10^4^ CFU∙mL^− 1^) for 2, 4, 8, 12, or 24 h compared to untreated controls. qPCR analysis showed that *S.c* induces SBD-1 mRNA expression in a time- and concentration-dependent manner. **b** Ruminal epithelial cells treated with *S.c* (5.2 × 10^8^, 5.2 × 10^7^, 5.2 × 10^6^, 5.2 × 10^5^, 5.2 × 10^4^ CFU∙mL^− 1^) for 2, 4, 8, 12, or 24 h compared to untreated controls. ELISA showed that expression of SBD-1 protein was consistent with that observed for SBD-1 mRNA. All experiments were repeated at least 3 times. *P < 0.05, **P < 0.01 vs. the control group

In the current study, results of qPCR indicated that *S.c* promoted SBD-1 mRNA transcription in a time- and dose-dependent manner (Fig. 2a). When the concentration of *S.c* was constant, the induction of SBD-1 was shown to be time-dependent. The maximal amount of SBD-1 mRNA was expressed after 12 h of incubation and decreased markedly after 24 h of incubation. When the incubation time was constant, the expression of SBD-1 mRNA was highest when cells were stimulated with 5.2 × 10^7^ CFU∙mL^− 1^ *S.c*, and was significantly higher than compared with cells stimulated with other *S.c* concentrations and compared to the control group (*P* < 0.01). Thus, the expression of SBD-1 was highest after incubation with 5.2 × 10^7^ CFU∙mL^− 1^ *S.c* for 12 h.

### *S.c* induced SBD-1 protein expression

The ELISA results demonstrated that the expression of SBD-1 at the protein level was consistent with that observed at the mRNA level (Fig. 2b). After the ovine ruminal epithelial cells were induced with various concentrations of *S.c* for different incubation periods, the SBD-1 protein expression in the co-culture supernatants was significantly higher compared to the control cells (*P* < 0.01). In short, the highest SBD-1 expression levels were achieved after a 12 h incubation with 5.2 × 10^7^ CFU∙mL^− 1^ *S.c*, which suggesting that these are the optimal *S.c* concentration and incubation time for SBD-1 induction. Therefore, an *S.c* concentration of 5.2 × 10^7^ CFU∙mL^− 1^ and an incubation time of 12 h was used to investigate the signalling pathways involved in the *S.c*-induced up-regulation of SBD-1 expression.

### mRNA expression levels of putative factors involved in *S.c*-induced up-regulation of SBD-1 in ruminal epithelial cells

**Fig. 3:**
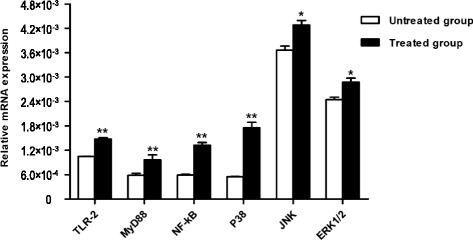
*S.c* stimulates expression of TLR2, MyD88, NF-kB, p38, JNK, and ERK1/2 mRNA as measured with qPCR. *P < 0.05, **P < 0.01 vs. the untreated group

To investigate the possible roles of TLR2, MyD88, p38, ERK1/2, JNK, and NF-κB activation in *S.c*-induced SBD-1 expression, ovine ruminal epithelial cells were stimulated with *S.c* and mRNA expression of the above factors was examined. As shown in Fig. 3, *S.c* treatment resulted in significantly increased NF-κB, p38, JNK, ERK1/2, TLR2, and MyD88 mRNA expression compared to the untreated cells (*P* < 0.05). The increased expression of these molecules suggests a putative role for these factors in *S.c*-mediated SBD-1 upregulation in ovine ruminal epithelial cells.

### MAPKs and NF-κB pathways were essential for *S.c*-induced up-regulation of SBD-1 expression

**Fig. 4:**
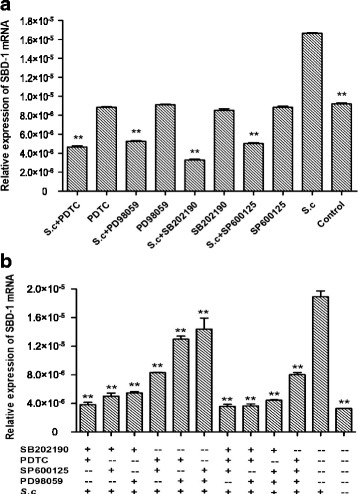
The role of MAPKs and NF-κB in *S.c*-induced SBD-1 mRNA expression. **a** Effect of different signalling pathway inhibitors on SBD-1 mRNA expression induced by *S.c*. Ruminal epithelial cells were cultured with *S.c* (5.2 × 10^7^ CFU∙mL^− 1^), with or without NF-κB inhibitor (PDTC), ERK 1/2 inhibitor (PD98059), p38 inhibitor (SB203580) or JNK inhibitor (SP600125) for 12 h. Total RNA was isolated, reverse-transcribed to cDNA, and the expression of SBD-1 mRNA was quantified with qPCR using specific primers for SBD-1 and β-Actin. **b** Effect of a combination of different inhibitors on *S.c*-induced SBD-1 mRNA expression. Ruminal epithelial cells were cultured with *S.c* (5.2 × 10^7^ CFU∙mL^− 1^), with or without NF-κB inhibitor (PDTC), ERK 1/2 inhibitor (PD98059), p38 inhibitor (SB203580), and JNK inhibitor (SP600125) for 12 h. Total RNA was isolated, reverse-transcribed to cDNA, and SBD-1 mRNA expression was measured using qPCR and specific primers for SBD-1 and β-Actin. All experiments were repeated at least 3 times. *P < 0.05, **P < 0.01 vs. positive controls

The inhibition tests demonstrated that MAPKs and NF-κB pathways are essential for *S.c*-induced up-regulation of SBD-1 expression (Fig. 4a). *S.c* significantly up-regulated the expression of SBD-1 mRNA (*P <* 0.01) in the positive control compared to the blank control group. Treatment with the specific NF-κB inhibitor, PDTC, significantly inhibited the expression of SBD-1 mRNA (*P* < 0.01) in the treatment groups compared to the positive control group. Furthermore, treatment with the specific inhibitors PD98059, SB202190, or SP600125 (MAPKs pathway inhibitors) also significantly inhibited the up-regulation of SBD-1 by *S.c* (*P* < 0.01). No significant difference was observed between the negative control group and the blank control group (*P* > 0.05). These results indicate that *S.c* can induce the up-regulation of SBD-1 expression through the NF-κB and MAPKs pathways. The 4 inhibitors decreased SBD-1 mRNA to different degrees with SB202190 having the biggest inhibitory effect. The effect of the 4 inhibitors was as follows: SB202190 > PDTC > SP600125 > PD98059. The effect of SB202190 was significantly higher than PDTC (*P* < 0.01), however the effect of PDTC was not significantly different compared to SP600125 or PD98059 (*P* > 0.05). These results suggest that the NF-κB and MAPKs pathways mediate the *S.c*-induced up-regulation of SBD-1 expression and that p38 in the MAPKs pathway may constitute a key signalling axis.

To further investigate the potential interaction between the NF-κB, p38, JNK, and ERK1/2 pathways, the 4 inhibitors were applied to the cells in combination. Groups of ovine ruminal epithelial cells were treated with various combinations of the inhibitors and then stimulated with *S.c* as follows: *S.c* + SB202190 + PDTC, *S.c* + SB202190 + SP600125, *S.c* + SB202190 + PD98059, *S.c* + PDTC+SP600125, *S.c* + PDTC+PD98059, *S.c* + SP600125 + PD98059, *S.c* + SB202190 + PDTC+SP600125, *S.c* + SB202190 + PDTC+PD98059, *S.c* + SB202190 + SP600125 + PD98059, or *S.c* + PDTC+SP600125 + PD98059. Cells treated with *S.c* alone served as a positive control group and cells not treated served as a blank control group (Fig. 4b). *S.c* significantly up-regulated SBD-1 expression (*P <* 0.01) in the positive control compared to the blank control group. In addition, when cells were treated with various combinations of the inhibitors, the *S.c*-mediated induction of SBD-1 mRNA was significantly reduced (*P* < 0.01) in treated groups compared to the positive control group. SB202190, the specific inhibitor of p38, had the most pronounced inhibitory effect.

## Discussion

Defensins are normal antibacterial peptides that act as important components of innate immunity in many organisms. In recent years, the role of probiotics in the regulation of β-defensin expression in the gastrointestinal tract of mammals has garnered a lot of attention. The relationship between probiotics and the expression of β-defensin genes in the gastrointestinal tracts of mammals has largely been investigated in Caco-2 human intestinal epithelial cells, in which inducible defensin expression by *Lactobacillus* and probiotic *Escherichia coli* was identified [45, 46]. However, few studies have been done on the relationship between fungi (such as *S.c*) and the defensin expression.

At present, the broadly accepted view is that probiotics can induce the expression of antimicrobial peptides in mammalian intestinal epithelial cells. In studying human intestinal epithelial cells (Caco-2), Wehkamp et al. [47] and Schlee et al. [46] found that the interactions between *Lactobacillus* and other probiotics and epithelial cells can increase the defensin expression and improve the body’s natural immune functions. However, these studies focused on the expression of antimicrobial peptides in human colon cancer epithelial cells and not in normal intestinal epithelial cells. Clearly, there are some differences in the basic structure, metabolism and function between cancer epithelial cells and normal epithelial cells, thus the understanding of these processes in normal cells is limited. Gastrointestinal tract antimicrobial peptides are mainly derived from epithelial cells, but can also be derived from intestinal mucosal immune cells [48]. Moreover, the role of probiotics in the body is affected by many factors, including gastric intestinal pH value, digestive enzymes, and bile. These factors can affect the viability of probiotic bacteria and the integrity of cell wall components. Therefore, to avoid interference from these complex variables and the impact of intestinal symbiotic bacteria, the effect of the probiotic *S.c* on the expression of the SBD-1 gene was investigated in normal sheep rumen epithelial cells.

SBD-1 mRNA levels were detected to elucidate the relationship between the probiotic concentration, the induction time, and the defensin expression. The results suggest that *S.c*-induced SBD-1 gene expression in sheep ruminal epithelial cells is dose- and time-dependent and that the effect on SBD-1 gene expression decreased when the *S.c* concentration was too low or too high. The SBD-1 mRNA levels were highest after the rumen epithelial cells were induced for 12 h at an *S.c* concentration of 5.2 × 10^7^ CFU∙mL^− 1^. In 2008, Schlee et al. [46] found that stimulation of Caco-2 cells with different probiotic lactic acid bacteria induced the expression of human β-defensin-2 (HBD-2) and that there was a dose- and time-dependent relationship between the bacterial concentration and the defensin expression. The expression of HBD-2 increased with the increasing dosage of different probiotics and reached a peak level after incubation for 6 h. In 2012, Guanhong Li and colleagues [49] determined that the expression level of HBD-2 mRNA peaked after the cultured chicken small intestinal epithelial cells were induced by *Lactobacillus rhamnosus LGA* at a concentration of 2 × 10^6^ CFU∙mL^− 1^ for 12 h. In 2014, Gácser et al. [50] showed that *Candida* spp. could induce the expression of HBD-2 in Caco-2 cells, with *Candida albicans* inducing the highest expression levels. Similarly, α-defensin (human neutrophil peptides, HNP 1–3) secretion was significantly increased in human whole blood after exposure to *Candida* yeast cells with *C. albicans* producing the greatest effect. Although there were differences in the concentration of bacteria and the incubation time, which resulted in the highest levels of the antimicrobial peptides, these differences may be species-specific and dependent on the particular stimulating components.

In the current study, ELISA was used to investigate SBD-1 expression at the protein level. The results showed that the expression of SBD-1 at the protein level was consistent with that observed at the mRNA level. Therefore, the *S.c*-stimulated expression of SBD-1 in ruminal epithelial cells was significantly higher than in the control group at both the RNA and protein levels, suggesting that SBD-1 expression is transcriptionally regulated. However, SBD-1 gene expression levels were not consistent at the mRNA and protein levels after the ruminal epithelial cells were cultured with 5.2 × 10^7^ CFU∙mL^− 1^ *S.c* for 2 or 24 h. This suggests that the expression of SBD-1 protein in ruminal epithelial cells may also be regulated post-transcriptionally, in addition to being regulated at the transcriptional level. The specific regulatory mechanisms require further investigation.

The results of this study indicate that the probiotic *S.c* can induce the expression of the SBD-1 gene in sheep rumen epithelial cells. If this also occurs in vivo, it may be of physiological significance as SBD-1 up-regulation may provide immunostimulatory effects. Thus, if the probiotic *S.c* can increase the ruminal innate immune function by up-regulating the expression of SBD-1 gene in ruminal epithelial cells in sheep, this may confer an immune benefit to the animal. The secretion of SBD-1 was confirmed by ELISA in in vitro studies using sheep ruminal epithelial cells. If this also occurs in vivo, SBD-1 may have extracellular biological functions. In addition, antimicrobial peptides secreted by ruminal epithelial cells may have a multitude of immunomodulatory effects as intercellular signalling molecules.

It is well-established that TLRs act as natural immune recognition receptors for the initial detection of microbes and serve as important bridges to activate the adaptive immune response [51]. It has been reported that probiotics are mainly recognized by TLRs in the intestin, and are involved in producing a prebiotic immune effect via the TLRs-mediated NF-κB signalling pathway. This interaction plays a crucial role in the maintenance of intestinal microbial and mucosal immune homeostasis [52]. Several studies have also confirmed that the peptidoglycan and lipoteichoic acid on the cell walls of beneficial bacteria can recognize the TLRs in epithelial cells and activate the downstream NF-κB signalling pathway [53, 54]. There is also evidence that the yeast, zymosan, activates TLR2/TLR6 heterodimers, whereas *S.c*- and *C. albicans*- derived mannan seems to be detected by TLR4 [55]. Glyc101 is a β-glucan isolated from *S.c*, Glyc101-induced immunomodulatory effector molecule production has been observed to be mediated via TLR-2 and NF-κB activation [56]. Based on this knowledge, the possible signal transduction mechanisms governing *S.c*-induced up-regulation of SBD-1 expression in ovine ruminal epithelial cells were investigated. It was found that the up-regulation of SBD-1 gene expression by *S.c* can affect the expression of TLR2 and MyD88 mRNA. This suggests that SBD-1 gene expression induced by *S.c* may be mediated by TLR2- and MyD88-dependent pathways. SBD-1, as an immune signalling molecule, can regulate the rumen mucosal immune response, thereby enhancing the animal’s immune system.

Recent studies have confirmed that the NF-κB pathway mediates the expression of some antimicrobial peptides in the intestines of mammals, including humans [57]. However, the expression of defensins is not only regulated by the NF-κB pathway, but also by MAPKs pathways. The expression of HBD-2 in oral mucosa epithelial cells can be induced by *Fusobacterium nucleatum* via the p38 and JNK components of the MAPKs signal transduction pathways [58]. Studies have also shown that the induction of defensin expression is co-mediated by the NF-κB and MAPKs pathways. Zhu et al. [59] reported that the release of mBD-14 by mouse osteoblasts was mediated by the activation of p38 MAPK and NF-κB in response to *S. aureus*-secreted bacterial exoproducts. Jia et al. [60] also confirmed that *Lactobacillus rhamnosus MLG*_*A*_, and its cell wall component, peptidoglycan, induced AvBD9 gene expression in chicken intestinal epithelial cells and that this was mediated by the TLR2-NF-κB/AP-1 signalling pathway, where TLR2-NF-κB constituted the main signalling axis. Results of this study showed that *S.c* up-regulated NF-κB and MAPKs signalling pathway molecules (NF-κB, p38, JNK and ERK1/2) at the mRNA level. Four specific inhibitors were used to elucidate the signalling pathways involved in *S.c*-induced up-regulation of SBD-1 expression and the results demonstrated a pronounced inhibition of SBD-1 mRNA levels following treatment with any of the 4 inhibitors, either alone or in combination, prior to *S.c* stimulation. Furthermore, SB202190, a well-characterised chemical inhibitor of p38 [61], was the most effective inhibitor of SBD-1, suggesting that while the NF-κB and MAPKs pathways may mediate the SBD-1 expression induced by *S.c*, the p38 axis may act as the main signalling mechanism. Therefore, signalling pathways regulating defensin expression induced by probiotics are likely not identical. This may be due to differences in the stimulating components of the specific species, but the explicit reasons require in-depth, follow-up study. At the same time, the results of this study showed that the 4 inhibitors could hinder the expression of SBD-1 only when the ruminal epithelial cells were stimulated by *S.c*. It may be that the expression of SBD-1 induced by *S.c* may occur through TLR2 membrane receptor ligation with the effective stimulating factor(s) of *S.c*, subsequent signalling to the kinase complex by the adapter protein MyD88, activating the NF-κB and MAPKs signalling pathways, resulting in the transcription of the SBD-1 gene by a variety of other coordinating proteins.

## Conclusion

In conclusion, *S.c* can increase the expression of SBD-1 in sheep ruminal epithelial cells. The SBD-1 expression levels were highest when the rumen epithelial cells were induced with *S.c* for 12 h at a concentration of 5.2 × 10^7^ CFU∙mL^− 1^. The regulatory mechanisms controlling *S.c*-mediated up-regulation of SBD-1 expression may function through the activation of the TLR2 and MyD88-dependent pathway and the downstream MAPKs and NF-κB pathways. Thus, this work suggests that the pathways involved in *S.c*-induced SBD-1 expression may be related to TLR2-MyD88-NF-κB/MAPKs signalling and that the TLR2-MyD88-p38 axis of the TLR2-MyD88-MAPKs signalling may act as the main pathway.

## Abbreviations

ELISA: enzyme-linked immunosorbent assay
MAPKs: mitogen-activated protein kinases
MyD88: myeloid differentiation factor 88
NF-κB: nuclear factor kappa-light-chain-enhancer of activated B cells
qPCR: quantitative real-time PCR
*S.c*: *Saccharomyces cerevisiae*
SBD-1: sheep β-defensin-1
TLR2: Toll-like receptor 2
